# Early intervention in schizophrenia

**DOI:** 10.4103/0019-5545.42402

**Published:** 2008

**Authors:** Parmanand Kulhara, Anindya Banerjee, Alakananda Dutt

**Affiliations:** Department of Psychiatry, Postgraduate Institute of Medical Education and Research, Chandigarh - 160 012, India

**Keywords:** Duration of untreated psychosis, early intervention, early psychosis, prodrome, schizophrenia

## Abstract

Early intervention (EI) programs in schizophrenia and other psychoses are aimed at early detection (ED) of the disease; prevent conversion to manifested psychosis and phase-specific treatment to reduce development of chronic disabilities. EI strategies include targeting people at “high risk" for developing schizophrenia, intervening in prodromal phase of schizophrenia, and reducing the “duration of untreated psychosis" (DUP). Services are delivered by a specialized team and are usually resource intensive. Several strategies like treatment with antipsychotics, family interventions, and cognitive behavior therapy have been tried with modest success in prodromal patients. Significant ethical reservations exist regarding exposing prodromal patients to the stigma of labeling as “high risk for schizophrenia" and side effects of psychotropics in the absence of clear evidence of efficacy in favor of ED, intervention by specialist teams, and phase-specific interventions in prodrome of psychosis. More research is warranted to demonstrate the risk-benefit and cost-benefit of such interventions before these can be routinely recommended.

## INTRODUCTION

Schizophrenia and other functional psychoses cause enormous suffering for individuals and their families. Despite new medications and community care, about one-third of people with schizophrenia have a poor long-term outcome. People with schizophrenia show a one-year relapse rate of 15-35%, rising to 80% within 5 years.[1,2] Achievement of full remission becomes less likely after each relapse and about 10% of sufferers eventually commit suicide. Even in developed nations like the United Kingdom, treatment is often delayed and it may take up to 2 years for families to seek help from first signs of psychosis.[3] Additionally, schizophrenia is a leading cause of health care expenditure, accounting for 2.5% (US $17.3 billion) of total health expenditure of USA, and in-patients accounted for 51% of total public sector expenditure on schizophrenia care.[4]

There are two approaches toward treatment of schizophrenia. In the orthodox approach, therapeutic resources are concentrated on people with established diagnosis. By the time help is sought, patients usually have severe and chronic disabilities. The new approach focuses on “early intervention” (EI) which involves greater investment of resources in the early stages of the disorder to reduce the number of people developing chronic disabilities. This notion is supported by the association between various outcome parameters and the duration of untreated psychosis (DUP).

## SCOPE OF ARTICLE

In this article, we discuss the components of EI---comprising early recognition in “high-risk” populations or in people with schizophrenia ‘prodrome’ and phase-specific treatment to reduce the DUP. We also review the efficacy and effectiveness of EI programs in schizophrenia.

## EARLY RECOGNITION

A key component of all EI is early recognition of the problem - ideally even before the emergence of clear-cut psychotic symptoms. There are two approaches to identify subjects who may manifest psychotic symptoms in later life: to target subjects who are at “high risk” to develop psychosis and those who display features of “prodrome” of schizophrenia.

### Early recognition: High-risk approach

#### Studies to identify high-risk groups:

These can be grouped under three categories:

Genetic/family studies
Birth cohort studies
High-risk projects

#### Genetic/family studies:

Genetic loading is known to be an important risk factor in schizophrenia and schizophrenia spectrum disorders. The lifetime risk of developing schizophrenia is about 10 times higher in first degree relatives of schizophrenia patients compared to the general population.[5]

#### Birth cohort studies:

Data from birth cohort studies indicate that low intelligence quotient at age 18, delayed motor milestones, motor co-ordination deficits, social and attention dysfunction in childhood, impaired social functioning, and behavioral abnormalities may predispose to higher risk of developing schizophrenia in later life. Other putative high-risk factors include exposure to prenatal/perinatal insults, obstetric complications, and maternal influenza.[6–8]

#### High-risk projects:

Some well-known high-risk studies include the Edinburgh high-risk study (EHRP) by Prof. Eve Johnstone and her group. The study began in 1994 and finished in 2004 thus completing 10-year period of observation. Other studies in this field are Copenhagen schizophrenia high-risk project initiated by Mednick and Schulsinger in 1962, the Israeli high-risk study by Mirsky and his group - a 25-year follow-up of high-risk children sponsored by National Institute of Mental Health (NIMH) of USA and the New York high-risk project (NYHRP) by Erlenmeyer-Kimling and his group which began in 1971.[9–11]

The findings from these studies indicate that those with genetic loading of schizophrenia, social withdrawal, social anxiety, schizoid and schizotypal personality, behavioral problems, and cognitive impairment are more likely to develop schizophrenia.[10]

Thus, these findings imply that individuals at enhanced-genetic risk of schizophrenia are a vulnerable group and may have transient and partial symptoms, more frequently than florid schizophrenia. It is possible, using simple behavioral assessments of schizotypal and anxiety-cognitions, to predict with some accuracy those of a high-risk group who will (and with considerable accuracy those who will not) develop schizophrenia, some years before the development of the psychosis.

The limitations of high-risk studies are that these studies refer to a group of individuals with a substantial family history of schizophrenia, who have been willing to participate in repeated assessments over prolonged period of time, and thus are not typical of the generality of individuals who may develop schizophrenia. Not all participants have passed through the principal period of risk of schizophrenia, and some who are currently well may yet develop psychosis. The control group volunteers are partly selected by their willingness to continue with prolonged ongoing studies, despite having no personal interest in the issue; they are, therefore, likely to be more socially responsible and persistent than average.

### Early recognition: Prodrome approach

The word prodrome has been derived from the Greek *prodromos* meaning precursor or forerunner. In psychiatry, it includes early symptoms and signs that precede characteristic manifestations of acute fully developed illness or relapse of schizophrenia.[12] Prodrome, therefore, may be as long as weeks, months, or years. The concept of prodrome in schizophrenia is not novel; equivalents of prodrome of schizophrenia have been described as minor changes of mood by Kraepelin, as latent schizophrenia by Bleuler and pseudoneurotic forms of schizophrenia by Hoch and Polatin.

The clinical features of the prodrome of schizophrenia are nonspecific and include nonpsychotic symptoms like early symptoms of depression and anxiety, irritability, insomnia, eccentric behavior, negative symptoms like apathy, social withdrawal, cognitive changes like inattention, impaired concentration and late symptoms like suspiciousness, ideas of reference, and perceptual abnormalities.[13,14]

The clinical features of the prodrome that are commonly associated with transition to psychosis are ideas of reference, unusual thought content, e.g., magical thinking; perceptual abnormalities, e.g., brief hallucinations, marked and rapid, functional decline, and social withdrawal.[15]

## STRATEGIES FOR IDENTIFYING PRODROME[14–16]

Although differences exist between the strategies, they all aim to minimize the identification of false-positives and to maximize true positive cases. These strategies include: “at-risk mental state,” basic symptom approach, and clinical high-risk approach.

High-risk or at-risk mental state strategy identifies those at high risk to develop schizophrenia. An individual must meet a number of conditions to be included in the high-risk group, e.g., behavioral difficulties in adolescence, social withdrawal, etc. as selection criteria. Unlike traditional screening paradigms, this approach is more clinically oriented, focusing on troubled young people, who are experiencing “precursor” signs and symptoms.

Ultra-high-risk strategy evolved by the Personal Assessment and Crisis Evaluation (PACE) Clinic, Melbourne, Australia includes adolescents with other potential risk factors like attenuated psychotic symptoms, self-resolving psychotic symptoms, a trait risk factor like schizotypal PD in the subject or a family history of a psychotic disorder and functional decline. The validity of this approach has been supported by longitudinal studies showing comparable rates of transition to psychosis from various centers.

The basic symptoms approach uses the Bonn Scale for the assessment of basic symptoms which is subtle, often only self-experienced deficits of several domains, like affect, perception, cognition, and social functioning. The Bonn long-term study found that 37% patients had these symptoms prepsychotically. The age-beginning course study not only confirmed the above, but also reported higher rates of these symptoms in patients.[17,18]

The clinical high-risk method is an extension of the Melbourne ultra-high-risk criteria for identifying young people thought to be at high risk of psychosis, also known as New York hillside recognition and prevention (NYH-RAP). The program recruited adolescents with specific combinations of cognitive, academic, and social impairments and disorganization/odd behavior (referred to as CASID features).[19]

To clinically determine schizophrenia prodrome, the DMS-III-R suggested a diagnostic criteria checklist that was tried by the American Psychiatric Association but was discarded by DSM IV due to lack of reliability. The search for better criteria continued, and the most widely accepted diagnostic criteria [Table 1][12] define three prodromal subgroups, namely attenuated positive symptom syndrome (APSS), brief intermittent psychotic syndrome (BIPS), and genetic risk plus functional deterioration (G/D).

In all three categories, frank psychosis (satisfying criteria of psychotic illness) must be ruled out.

Two most commonly used instruments for diagnosing prodromes are the comprehensive assessment of symptoms of at-risk mental states (CAARMS) and structured interview for prodromal syndromes (SIPS).[20] CAARMS is a structured diagnostic interview operationally defining a mental state at “'ultra-high-risk" for conversion to psychosis and is a part of the PACE project, Melbourne. This instrument was revised later and is now known as SIPS and is reported to have good reliability and predictive validity.[21]

## DIFFERENTIAL DIAGNOSIS OF PRODROME

The prodrome of schizophrenia is nonspecific and it may be extremely difficult to clinically distinguish it from a host of other disorders. Such prodromal symptoms are classically present in adolescents and are often confused with psychotic depression, schizotypal personality disorder, borderline personality disorder, autistic spectrum disorders, obsessive compulsive disorder with poor insight, post-traumatic stress disorder (PTSD), attention deficit hyperactivity disorder (ADHD), or simply an adolescent crisis which may not tantamount to any axis-I disorder.[12] It is extremely important to exercise great deal of caution before labeling any adolescent to have prodrome of schizophrenia for risk of stigmatizing that individual as attaching a label of schizophrenia, even though prodrome, may have disastrous consequences.

Over the last century, there has been a big leap in the concept of prodrome with change in terminology from “latent schizophrenia” to prodrome of schizophrenia. However, it still remains a diagnostic and management challenge, though some consensus is evolving as to what is to be regarded as prodrome, with the emergence of prospective diagnostic criteria for prodrome. An important issue in prodromal intervention is the ethical consideration, potential for false-positive and for false-negative identifications, and risk of being stigmatized by treatment or research participation.

## DURATION OF UNTREATED PSYCHOSIS

It is a topical and popular yet elusive concept. It denotes the time from manifestation of the first psychotic symptom to onset of antipsychotic treatment. However, it is limited by the absence of consensus about onset of psychosis or treatment and recall bias.[22]

The interpretation of the term “psychotic symptoms" may vary from any psychotic symptom to specified symptoms as measured by a scale while onset of treatment is defined variously as admission to a hospital, effective antipsychotic treatment or adequate period of treatment (>1–3 months).

**Table 1:** Prospective diagnostic criteria for prodromal syndromes (as given by PACE Clinic, Melbourne) - diagnosed using comprehensive assessment of symptoms of at-risk mental states (CAARMS)

Attenuated positive symptom syndrome (APSS)	Unusual thought content, suspiciousness, grandiosity, perceptual abnormalities, and/or formal thought disorder that does not satisfy frank psychosis; occurring at least once a week in last month; with worsening course in past 12 months
Brief intermittent psychotic syndrome (BIPS)	Clearly psychotic thought content, paranoia, grandiosity, perceptual abnormalities, and/or formal thought disorder; with onset within last 3 months
Genetic risk + functional deterioration (G/D)	History of any psychotic disorder in first-degree relative *OR* Schizotypal personality disorder in patient
	Associated with marked functional decline in the past year

Several studies have reported association between longer DUP and worse outcome at 6 months in terms of total symptoms, overall functioning, positive symptoms, and quality of life in patients with schizophrenia. Patients with a long DUP were significantly less likely to achieve remission.[23–25]

Given its relationship with outcome, DUP assumes significant public health importance as it is a potentially modifiable prognostic factor. It also has implications for understanding the pathophysiology of schizophrenia and may indicate toward a neurodegenerative process. However, the evidence in this regard is inconsistent.[26]

Structured assessment of DUP can be done by diagnostic tools like interview for the retrospective assessment of the onset of schizophrenia (IRAOS),[27] Royal Park multidiagnostic instrument for psychosis,[24] and Nottingham onset schedule.[28]

There are several limitations in applying the concept DUP for planning intervention programs. First, it is uncertain whether DUP is a determinant or a marker of outcome. The use of variable cut-offs across studies fails to distinguish between a “short DUP” and a “long DUP.” There is a possibility of it being confounded by personality and/or illness-related variables like age at onset, gender, premorbid functioning, socio-economic status and mode of onset, which may influence help-seeking behavior and delayed initiation of treatment; thus prolonging the DUP. Moreover, it is difficult to reliably determine the exact onset of symptoms, as assessment is retrospective and subject to recall bias.[22]

The most pertinent concern is whether DUP is a valid measure of effectiveness of intervention services. Should reducing DUP be the cardinal target of intervention programs? This question is not easily answered, given all the reservations presented above, which cast doubt on reliability of DUP as the sole predictor of outcome. Additionally, the evidence for the effectiveness of early detection (ED) programs in reducing DUP is equivocal. The available data do not support that establishment of specialized ED services has any overwhelming impact on outcome through reduction of DUP.[22]

## EARLY INTERVENTION

EI programs (EI) are those interventions aimed at ED to prevent the onset of schizophrenia in people who are likely to develop schizophrenia (primary prevention) as well as to provide effective treatment to people in the early stages of schizophrenia, with the goal of reducing the ultimate severity of the illness (secondary prevention).

EI differs from standard care on the basis of ED and phase-specific treatment being provided by specialized EI teams. EI and EI teams are expected to meet needs of people aged 14-35 with symptoms of psychosis for the first time and 14-35 year olds during the first 3 years of psychotic illness.[29]

The terms early intervention (EI) and early psychosis (EP) Programs are often used interchangeably. However, EI conventionally relates to schizophrenia and EP to other psychosis including schizophrenia. Most EI or EP programs are similar with minor variations or differences irrespective of the target groups (“at risk,” “prodrome,” or “DUP”).

## DEVELOPMENT AND GROWTH OF EI-EP PROGRAMS ACROSS THE WORLD

EI programs were first established in Melbourne, Australia and Buckinghamshire in the UK in mid-1980s and then extended to Birmingham (UK), Germany, USA, Canada, Scandinavia in early 1990s, Switzerland in mid-1990s, Amsterdam and Australia in late 1990s. In 1999/2000, EI in UK received a major boost following extensive funding from NHS. By 2001, it had spread to Far East and South East Asia.

**EI-EP service-models:** There are three models of EI-EP service delivery.

1. Enhancing existing community mental health teams – CMHT.
2. 'Hub and spoke' model: The hub is a central specialist service which supports mainstream services by providing specialist input into individual cases.
3. Standalone early intervention service

The advantages and disadvantages of each are summarized in Table 2.[30,31]

**Table 2:** Models of early intervention teams

Models	Advantages	Disadvantages
Community team model	Availability of more staff Complements existing structures Inexpensive	Lacks target and focus No new expertise involved
Hub and spoke model	Easier to establish	Perpetuates existing problems within services Has no independent identity
	Fewer resources needed	Interface responsibility problems
		No element of ED
Standalone model	Incorporates ED Evidence-based care Allows staff development	Resource intensive Potential for isolation Loss of continuity at discharge
	Evidence-based care	Potential for isolation
	Allows staff development	Loss of continuity at discharge

## EFFECTIVENESS OF EARLY INTERVENTION SERVICES (EIS)

### Effectiveness of EIS in “at high risk”

Genetic risk at present possibly cannot be modified, but genetic counselling may help to decrease genetic risk. Other putative factors like prenatal and perinatal obstetric complications, schizotypy, other behavioral and cognitive deficits, social anxiety, depression, and risk of suicide may also be modifiable.[10]

Intervention work in the EHRP which began recently has found some impact on suicide rate but rate of conversion to schizophrenia remains the same. These findings have been corroborated by the NYHRP and Hillside recognition and prevention (H-RAP). Preliminary RAP intervention findings suggest that medications other than anti-psychotics may be effective for treating early prodromal symptoms, challenging the widely held hypothesis that anti-psychotics should always be the first line preventive treatment.[32]

### Effectiveness of EIS in “prodrome””

Only few randomized controlled trials (RCTs) suggesting usefulness of EI in prodromal phase are available till date. In a RCT, McGorry *et al.*[33] compared combination treatment with intensive psychological (cognitive) treatment along with very low-dose risperidone medication with supportive therapy (need-based intervention) alone. After 6-month of treatment significantly more acute psychosis was seen in the supportive therapy group. On discontinuing risperidone, some of the patients went on to convert to psychosis. Thus, it might be possible to delay the onset of acute psychosis by the use of combined treatment.

In a double-blind, placebo-controlled clinical trial of patients in prodrome who were randomly assigned to either olanzapine or placebo for 12 months which was followed by a 12-month monitoring period. There was significant difference between the olanzapine and placebo group at 6 weeks.[34,35] In the same cohort, McGlashan *et al.* demonstrated that 16.1% of the olanzapine group and 37.9% of the placebo group converted to psychosis.[36]

Cornblatt *et al.*,[32] in a prospective, naturalistic treatment study of adolescents in prodromal phase compared the effect of antidepressants (*n* = 20) and second generation antipsychotics (SGA) (*n* = 28) on conversion rate. Twelve of the 48 adolescents (25%) developed a psychotic disorder; all the converters being in the SGA group. However, 11 of the 12 converters were nonadherent to SGA.

Thus, results so far are not very convincing, however, it may be premature to assume that EI will not fulfil its promise.

### Effectiveness of EIS in “DUP”

Experiences from a EI-EP study, The early treatment and intervention in psychosis (TIPS) in Stavanger, Norway has revealed that it is possible to shorten DUP from 114 to 20 weeks.[37]

Other studies aimed at reducing DUP, show ambiguous results and, until now, no follow-up data showing a positive effect on prognosis have been presented.[38]

A study from Denmark and Norway using ED strategies showed that DUP was reduced by 1.5 years (mean) from before the time the ED system was instituted (to 0.5 years). Thus, ED strategies of targeted information appear to be effective and influence directly the community's help-seeking behavior.[39]

Information campaigns (IC) directed toward general public, schools, and primary health care services which was the key factor in reducing DUP from 16 to 5 weeks in TIPS Program in Norway, when stopped, led to the loss of the advantage in reducing DUP.[40]

ED is central to most EI-EP programs, so early recognition will facilitate EI and decrease DUP. Evidence suggests that it is difficult to design studies that are both ethical and potent enough to determine the contribution of treatment delay to outcome.[41] Further research is justified but this should not obstruct common-sense service reforms.

A recent Cochrane Database meta-analytic review evaluated effects of ED, phase-specific treatments, and specialized EI teams in people with prodromes and first episode psychosis. Out of 65 EI studies evaluated, only seven RCTs with a total of 941 patients were found eligible for inclusion in the reviewed meta-analysis. Most RCTs were under-powered; and the authors concluded that the data were insufficient to draw any definitive inference. There was no clear evidence in favor of ED, intervention by specialist teams, and phase-specific interventions in first episode psychosis.[42] This meta-analytic review is advised caution in rushing in to establishing EI programs taking in to consideration the cost and resources involved.[42]

## ETHICAL ISSUES IN EI

There are key ethical considerations regarding treatment of nonpsychotic “prodromal” individuals thought to be at high risk for developing schizophrenia. Current estimates suggest that one in four such persons will convert to schizophrenia. Is it in the domain of medical profession to intervene in such cases, where clearly defined illness is absent and, in majority of cases, may not develop at all? If at all we intervene to modify the behavior, what should be the scope, aim, and duration of such interventions? One cannot ignore the risk of significant stigma carried by the label of a devastating mental illness like schizophrenia. Can the benefits of being labeled “at risk” for schizophrenia outweigh the risk of the stigma associated with it?[43]

Given the potential for false-positivity, is there a solid rationale for prescribing antipsychotics to people who at that time are not psychotic and exposing them to the long-term hazards of metabolic syndromes associated with such medications. On the contrary, if antipsychotics really prevent conversion to psychosis, placing a true positive on a placebo is unethical as well.[44]

EI programs require substantial resources but evidence of efficacy and effectiveness of EI is still questionable. Some authors suggested that in the recent years there has been an “ethical-paradigm shift" in favor of EI in psychosis.[45] However, effectiveness of these programs on account of risk-benefit as well as cost-benefit contentious.

## CONCLUSION

EI in schizophrenia involves ED and delivery of phase-specific treatments by a specialized team. It does not simply imply antipsychotic treatment to prevent conversion to psychosis or in early psychosis. For patients in prodrome of schizophrenia, frequent follow-up, treatment alliance, provision for crisis intervention, detailed assessment of functioning at baseline, and at intervals; address to safety issues and comorbidities are all important aspects of EI services. Other interventions like supportive therapy, family therapy, psychoeducation, and liaison services should also be offered as part of treatment package.

Although some data support benefits of treatment with antipsychotics prior to development of psychosis, it is still in research phase and not routinely recommended. Given insufficient and inconsistent evidence regarding risk-benefit and cost-effectiveness, it may be premature to implement wide-spread ED programs and EI team-based treatment programs for people with prodromal symptoms or early psychosis. However, treatment in manifested psychosis must be offered as soon as possible.
